# TME4/349: Teledermatology: Rural General Practitioner and Metropolitan Specialist Collaboration

**DOI:** 10.2196/jmir.1.suppl1.e111

**Published:** 1999-09-19

**Authors:** J Hovel

**Affiliations:** ^1^Monash UniversityTraralgon, VictoriaAustralia

**Keywords:** Clinical Decision Support Systems, Telemedicine, Remote Consultation

## Abstract

**Introduction:**

A major research report in 1995 identified a lack of dermatology services outside major cities in Australia. This paper reports the progress of a teledermatology trial using digital still photography and e-mail over Internet technology to connect rural family doctors with metropolitan specialists for advice and support, normally available only through referral and travel by the patient. Teledermatology is an application of information exchange techniques to perform dermatology consultations at a distance from the patient. In most cases, some or all of these functions are performed by a family doctor face-to-face with the patient and relayed to a specialist. In many cases, the confirmation of diagnosis and treatment plan by the specialist is all that is required. The patient will be spared referral and often extensive travel and the rural family doctor will be supported in their assessment and treatment of skin disorders. Teledermatology can also result in clinical supervision for rural doctors. The project demonstrates that this technology is an acceptable and convenient alternative for specialists, rural family doctors and their patients to traditional referral methods.

**Methods:**

The project is a descriptive, prospective study of the technical feasibility and acceptability of store and forward teledermatology utilising digital images of skin conditions and Internet e-mail between rural family doctors and urban dermatologists. This trial links four rural Victorian family doctors to two Melbourne dermatologists. Patients selected by the family doctors on the basis of their clinical signs and symptoms are asked for their consent to participate in the project. Their conditions are non-urgent and unclear to their rural doctor in either diagnosis or management. The resulting consultation by dermatologists clarifies diagnoses and management, or may result in physical referral to the dermatologists. In many cases, punch biopsies confirm the validity of the diagnoses made by either family doctor or dermatologist (as is the current clinical practice).

**Results:**

The current phase of the project provides technical evaluation, educational support, specificity and reliability testing and instruments validation. During this phase, suitable reporting mechanisms and hardware and software configurations are being identified and implemented. Testing of the techniques and protocols thus far indicates that the currently available technology is suitable for the tasks proposed and is acceptable to patients, family doctors and dermatologists.

**Discussion:**

The experience and data collected will provide the foundation for a much larger study which will enable generalisation of results to rural areas in Australia and internationally.

